# Significance of immunohistochemistry and FISH of TFE3 in the diagnosis of alveolar soft part sarcoma: A case report

**DOI:** 10.1097/MD.0000000000029861

**Published:** 2022-07-08

**Authors:** Yan-Ying Huang, Wan-Rui Yang, Yan-Hua Geng, Yue Zhang

**Affiliations:** a Department of Pathology, Hangzhou Red Cross Hospital, Hangzhou, Zhejiang, People’s Republic of China; b Department of Radiology, Hangzhou Red Cross Hospital, Hangzhou, Zhejiang, People’s Republic of China.

**Keywords:** ASPS, brain, IHC, TFE3

## Abstract

**Rationale:**

Alveolar soft part sarcoma (ASPS) is a rare soft tissue sarcoma harboring an *ASPL-TFE3* fusion gene. Herein, we report a case of ASPS associated with brain metastasis. Immunohistochemistry (IHC) for TFE3 antigen expression and fluorescence in situ hybridization (FISH) for *TFE3* rearrangement were performed to arrive at an accurate diagnosis.

**Patient concerns:**

A 47-year-old man was hospitalized for a headache and numbness of the lower limbs.

**Diagnoses:**

Preoperative computed tomography and magnetic resonance imaging revealed 2 brain masses, 1 each in the right parietal and temporal bones. We diagnosed this case as ASPS with brain metastasis based on histological morphology, IHC, and FISH.

**Interventions:**

The patient underwent right skull titanium mesh implantation and supratentorial superficial lesion resection.

**Outcomes:**

: The patient recovered well after discharged from hospital.

**Lessons:**

The diagnosis of ASPS depends on careful clinical, radiographic, histopathological, IHC, and FISH assessments to arrive at the correct diagnosis. Thus, TFE3 may be useful in the diagnosis and treatment of ASPS.

## 1. Introduction

Alveolar soft part sarcoma (ASPS) is a rare and malignant soft tissue sarcoma that accounts for only 0.5% to 1.0% of soft tissue tumors.^[1–2]^ ASPS was first discovered and described by Christopherson et al.^[3]^ This type of tumor is more common in young people, particularly those aged 15 to 35 years.^[4–7]^ ASPS usually affects the head and neck of children.^[4]^ However, they usually occur in the lower limbs of adults.^[5]^ The main characteristics of the tumor are its insidious onset, which manifests as a painless, asymptomatic mass in the deep parts and rapid metastasis. ^[8]^ Metastatic tendency of ASPSs is high, and some patients have metastatic disease at the time of hospital visit. Metastasis occurs primarily in the lungs (42%), bones (19%), brain (15%), and lymph nodes (7%).^[9,10]^ The prognosis for patients with metastasis is poor. A nonreciprocal chromosomal translocation der(17)t(X;17)(p11;q25) of ASPS results in an *ASPSCR1-TFE3* gene fusion. Therefore, targeted therapy may be an effective treatment option for ASPS.^[11]^ Herein, we report a case of ASPS with brain metastasis and analyze it via immunohistochemistry (IHC) and fluorescence in situ hybridization (FISH).

## 2. Case report

A retrospective chart review of the patients was conducted at Hangzhou Red Cross Hospital after obtaining approval from the institutional review board. A 47-year-old man was referred to our hospital with headaches and numbness of the lower limbs for 7 days. He had a history of lumbar tuberculosis for 4 years. Specialist examination: the right occipital lobe reached a 3 × 3 cm mass, similar to the right temporal-frontal lobe.

**Figure 1.**
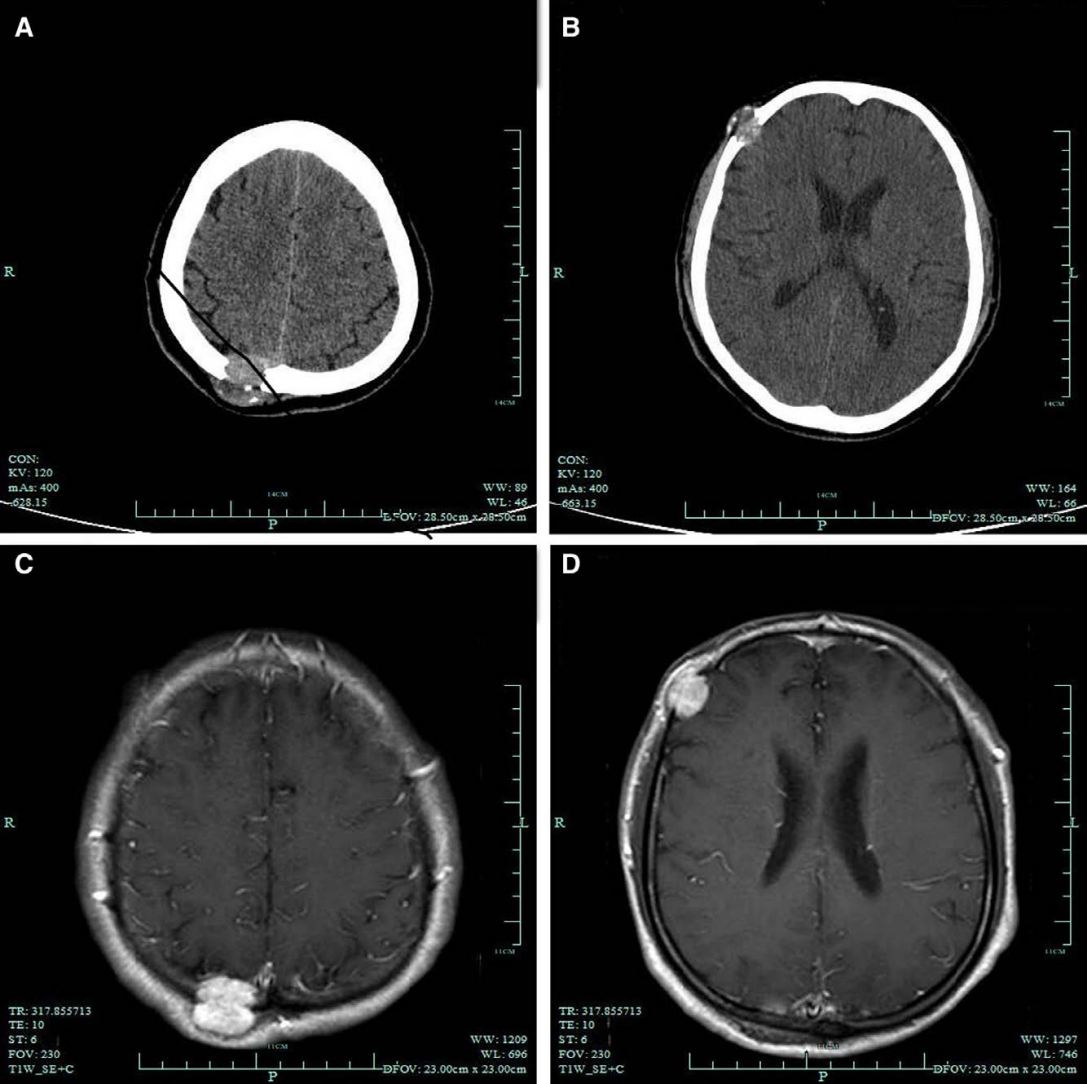
The detection results of CT and MRI. (A) CT scan showing right parietal bone destruction with soft tissue shadow. (B) Computed tomography scan showing right temporal bone destruction with soft tissue shadow. (C) T1-weighted image of the right parietal bone showing hyperintensity. (D) T2-weighted image of the right temporal bone showing hyperintensity.

Computed tomography and magnetic resonance imaging revealed 2 brain masses, 1 each in the right parietal and temporal bone. Computed tomography of the brain showed destruction of the right parietal and right temporal bones with soft tissue shadow (Fig. 1A and B, respectively), and the right parietal, temporal, and right frontal bones were locally damaged with soft tissue shadows, and metastatic tumors were considered. Magnetic resonance imaging of the head revealed multiple nodular signal shadows on the skull of the right parietal-temporal lobe. T1- and T2-weighted hyperintensities were observed in the right parietal and temporal bones, respectively (Fig. 1C and D). Therefore, right-sided soft paraventricular lesions should be considered.

**Figure 2.**
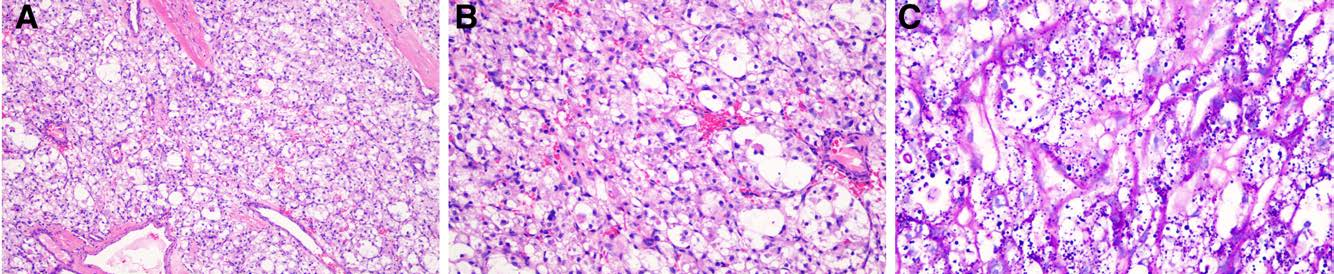
Histologic features and PAS staining of ASPS. (A) The cells were arranged in an acinar or nest-like pattern (H&E; ×100). (B) Tumor nuclei were often vesicular or polygonal with significantly enlarged nucleoli (H&E; ×400). (C) PAS-positive acicular or rod-like crystals in the cytoplasm. H&E = hematoxylin and eosin, PAS = periodic acid–Schiff.

**Figure 3.**
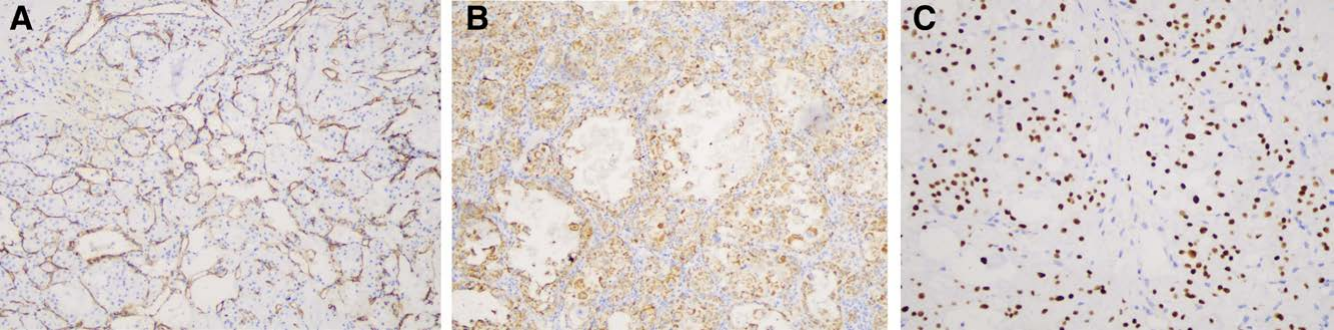
Immunohistochemical analyses. (A) CD34 was negative in tumor cells but positive in the wall of the blood between the alveolar structures. (B) MyoD1 expression in tumor cell cytoplasm. (C) Tumor cells showed diffuse and strong nuclear positivity for TFE3 revealed by immunohistochemistry.

**Figure 4.**
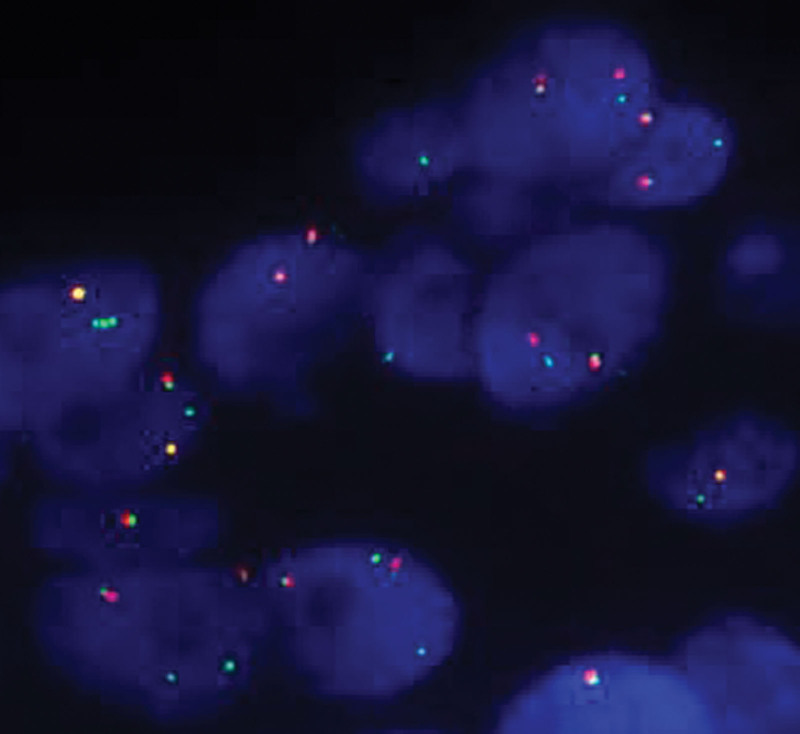
*TFE3* split FISH assay showed green and red split signals, indicating *TFE3* gene rearrangement. FISH = fluorescence in situ hybridization.

Subsequently, the patient underwent right skull titanium mesh implantation and supratentorial superficial lesion resection, and a 2 × 2 × 2 cm mass was observed in the forehead. Blood supply to the tumor was abundant. Microscopically, the tumor cells were arranged in an acinar and nest-like pattern, and the nests tended to be uniform in size and shape. The nests were separated by delicate sinusoidal vascular channels lined by a flattened single layer of endothelial cells (Fig. 2A). The tumor nuclei were often vesicular or polygonal, with significantly enlarged nucleoli (Fig. 2B). Periodic acid–Schiff staining showed that the intracytoplasmic glycogen and characteristic periodic acid–Schiff-positive cytoplasm of the tumor cells were red and finely granular with red-dyed rod-shaped crystals inside (Fig. 2C). IHC analysis of CD34 highlighted the delicate capillaries surrounding tumor cells or nests (Fig. 3A). IHC analysis revealed that the tumor cells were cytoplasmic positive for MyoD1 (Fig. 3B) and nuclear positive for TFE3 (Fig. 3C). These results narrow the differential diagnosis of ASPS. A FISH assay was performed to confirm the diagnosis. The results showed green and red split signals, indicating TFE3 rearrangement (Fig. 4). Thus, a final diagnosis of ASPS with multiple brain metastases was made, and after 6 months of follow-up, no evidence of recurrence was observed.

## 3. Discussion

ASPS is a rare soft tissue tumor that harbors the *ASPL-TFE3* fusion gene. These tumors are most often observed in deep soft tissues of the extremities. ASPS usually presents as a slow-growing tumor without pain and is characterized by early metastasis, which leads to poor prognosis. The most common metastatic sites are the lung, bone, and brain; unlike most sarcomas, metastases to the lymph nodes are uncommon,^[12–13]^ which may precede the detection of the primary tumor.^[14–15]^ When some ASPS grow in rare locations, the pathological morphology of partial ASPS is atypical.

TFE3 is a useful immunohistochemical marker for the diagnosis of ASPS. Most ASPS samples were positive for TFE3 by IHC, with strong nuclear staining. Our ASPS demonstrated strong (3+) labeling and strong nuclear immunoreactivity for the TFE3 protein, which proved to be highly sensitive and highly specific for neoplasms with TFE3 fusion proteins.^[16]^ However, it has limited specificity as it is also expressed in a granular cell tumor, Xp11.2 translocation-positive renal cell carcinoma, adrenocortical carcinoma, and in 10% of perivascular epithelioid cell tumors.^[13,17]^ In addition, it is technically difficult to detect TFE3 reactivity using IHC, as it is not accompanied by a strong background stain or even false-positive and false-negative results. Thus, although IHC is highly sensitive for tumors associated with *TFE3* gene fusion, a negative result may not completely exclude a diagnosis. Recent studies have shown that molecular analysis, such as FISH for TFE3 gene rearrangement, is a powerful tool for the diagnosis of ASPS.^[18–19]^ This suggests that TFE3 by IHC plays only a minor role in the diagnosis of *TFE3*-rearranged tumors because FISH is the preferred method.

ASPS is characterized by an unbalanced translocation that results from the fusion of the *TFE3* gene at Xp11 to the *ASPL* gene at 17q25, resulting in fusion of the *TFE3-ASPL* gene.^[20]^ Reverse transcription-polymerase chain reaction and FISH are tools available for identifying this translocation. However, reverse transcription-polymerase chain reaction may be limited by RNA quality due to the degradation of RNA in the archival material. FISH is a cost- and time-efficient method that uses the most accessible material in the laboratory, formalin-fixed, paraffin-embedded tissues, for the detection of *TFE3* (Xp11.2) gene rearrangement. FISH has been shown to be a highly sensitive and specific test for *TFE3* rearrangements.^[21–23]^ In this study, we used a break-apart FISH probe assay and observed green and red split signals, indicating *TFE3* gene rearrangement. This finding further confirms the diagnosis of ASPS.

ASPS is extremely rare, and its prognosis is poor, often characterized by late metastases. Some studies have shown that the most important parameters for prognosis are age at diagnosis, tumor size, and presence of metastasis. Younger age at presentation and smaller tumor size are associated with better tumor prognosis.^[24]^ That is, there is a significant relationship between tumor size and the probability of metastasis in ASPS. It has been reported that following surgical resection, the 5-year survival rate of patients without metastasis is 87%, while that of patients with metastasis is only 20%.^[25]^ The patient was a 47-year-old man with a small tumor measuring 2 cm with free surgical margins. The patient had no signs of recurrence or disease 6 months after surgery.

In conclusion, TFE3 immunostaining and fusion gene *ASPL-TFE3* expression may provide information for clinical molecular pathological diagnosis and improve the diagnostic rate of ASPS. Patients should be strictly followed up after surgery to detect residual/recurrent disease and delayed metastases.

### Acknowledgments

The authors thank Editage (http://www.internationalscienceediting.com) for editing the manuscript.

### Author contributions

**Writing – original draft:** Yan-Ying Huang.

**Writing – review & editing:** Yan-Ying Huang, Yue Zhang.

**Conceptualization:** Wan-Rui Yang.

**Data curation:** Yan-Hua Geng, Yan-Ying Huang, Yue Zhang.

**Funding acquisition:** Yan-Ying Huang.

**Investigation:** Wan-Rui Yang,Yan-Hua Geng.

**Resources:** Yan-Ying Huang, Yan-Hua Geng, Yue Zhang.

**Supervision:** Yue Zhang.
